# Trains scheduling problem with multiple lines

**DOI:** 10.1038/s41598-024-82499-0

**Published:** 2024-12-28

**Authors:** Venkata Prathap Danavulapadu, Purusotham Singamsetty

**Affiliations:** Department of Mathematics, School of Advanced Sciences, Vellore Institute of Technology, Vellore, Tamil Nadu 632014 India

**Keywords:** Train scheduling, Routing, Branch and bound algorithm, Zero–one integer programming, Engineering, Mathematics and computing

## Abstract

This study explores the problem of train scheduling (or) train timetabling and its impact on the administration of railway management. This is a highly dependable and effective public transportation system. The problem considers both single and multiple tracks along with multiple platforms with varying train capacities (like speed, passengers, and so on). Let the tracks link two major stations source and destination with intermediate stations. A subset of intermediate stations will function as junctions. A finite number of trains be available at the source for the passengers/cargo transit movements. The profit which is generated from the shipment of passengers between a pair of stations is known in advance. The train’s travel time between the stations and halting time at the stations are predefined. The arrival and departure times at the stations are calculated through the travel time and halting time. All the trains should starts from the source station and continue the journey through the intermediate stations to reach the destination station. Overtaking of the trains is permitted only at the intermediate stations. The trains must make a halt at one or more intermediate stations before reaching the destination. Now the objective is to find the best train operating schedule that maximizes the profit within the admissible travel time threshold. A zero–one integer linear programming is used to model this problem mathematically. For a better understanding of this problem, a case study is considered from the Indian railway network with two major stations Chennai and Hyderabad. A branch and bound $(B\&B)$ algorithm is proposed to determine an optimal operating schedule. In addition, the experiments are carried out on a wide range of randomly generated instances of small and medium sizes, to test the efficiency of the algorithm. The computational results indicate that the algorithm is capable of finding the optimal schedules within a reasonable amount of time.

## Introduction

Public rail transportation involves several stages, which include analyzing passenger demand, determining routes, scheduling trains, planning rolling stock, and managing crews. This paper focuses on the train scheduling problem$/$train timetabling problem (TTP) and it is related to combinatorial optimization. In general, the rail network system connects distinct places termed as stations with multiple rail tracks. To be precise, the stations and the rail track$/$connectivity between a pair of stations are referred as the nodes and arcs of a network respectively. On each arc, a weight factor such as travel time, profit, distance, cost, etc. may be defined. The TTP determines an optimal train schedule for the route plan, stopping plan, arrival and departure times. This guarantees a smooth travel experience for passengers and helps to prevent any conflicts among the different trains.

The literature shows a significant amount of research carried out on the TTP in the last one to two decades due to the high utilization of passenger movements. The early works of TTP can be found in Szpigel^1^, in which each pair of stations are connected by a track section, permits the presence of only one train at a given time on this single-track line and aims to minimize the weighted average of the train travel times. A mixed integer linear programming model is used to express the TTP mathematically and solved with the B&B method. The algorithm carefully resolves the train conflicts with the effective lower bounds. However, the computational results are limited to small size instances.

Jovanovic and Harker^2^ considered this TTP for the tactical scheduling of the freight rail road traffic which supports the weekly or monthly train scheduling operations with an aim of minimizing the deviation between the planned and the actual train schedules. In order to model the train movements and interactions over the given track, a deterministic process interaction simulation technique was incorporated in the B&B algorithm. Each level of the search tree mechanism used in the B&B represents the resolution of conflict between a pair of trains and the nodes indicate the potential meet points to resolve the conflicts. The algorithm is capable of providing an overview of the speed limits on the single track section.

Cai and Goh^3^ gave an example of finding a feasible solution to the TTP with 12 passing loops, solved through a greedy heuristic approach. Higgins et al.^4^ investigated the single line TTP with the aim of resolving conflicts between the trains and suggested different heuristic approaches such as local search, genetic algorithm, and tabu search. The algorithms tested on the different instances with the number of trains ranging from 15 to 50. The computational results shows GA is producing promising solutions within the reasonable amount of time than the other tested heuristics.

Oliveira and Smith^5^ modeled the TTP as a special case of the job-shop scheduling problem. The problem arises when two trains arrive at the same track section at the same time, conflicts are happen and resolved by adjusting the timings of the trains. The objective is to minimize the delay of trains. A constraint programming approach is proposed and experimented on some real-life problems. Caprara et al.^6^ studied the TTP with a single track that connects two major stations with a finite number of intermediate stations. The problem is nicely interpreted in a graph theoretic view in which nodes represents the arrival/departures at some station within the given time instant. An integer linear programming is used to model this TTP and solved with the help of the Lagrangian relaxation technique. The algorithm tested on the instances acquired from Ferrovie dello Stato Spa, the Italian railway company, and obtained good solutions with in a short computational time.

Ghoseiri et al.^7^ developed a multi-objective optimization model for the TTP that considers both single and multiple tracks along with multiple platforms with varying train capacities. One objective is reducing fuel consumption and another objective is minimizing the passenger’s journey time. Numerical examples are provided and solved through two approaches: the first approach involves identifying the Pareto frontier utilizing the e-constraint method, while the subsequent approach involves performing multi-objective optimization through the distance based method.

An interesting variant of the TTP was studied by Vansteenwegen and Van Oudheusden^8^ with a focus on reducing the waiting times of passengers and late arrivals of trains to improve the passenger service. A two phase linear programming approach was applied to the Belgian railway network. In the first phase the ideal buffer times were calculated and in the subsequent phase constructed an improved timetable with ideal buffer times. The delayness of the trains run time was improved up to 49% with the help of linear programming. Later in 2007 they^9^ extended this approach to the intercity network of Belgian railways consisting of 14 high speed trains connecting the main cities of Belgium. D’ariano et al.^10^ studied the TTP faced some difficulties during the traffic control. A $B\&B$ algorithm was developed and included some implication rules for computational experiments. Also presented a case study on the Dutch railway network and obtained optimal or near optimal solutions within a reasonable amount of time.

Some of the earlier works on TTP appeared on the issue of train delays can be found in Kroon et al.^11^ and Liebchen and Stiller^12^. Kroon et al.^11^ developed a stochastic optimization model that can be used to allocate additional time supplements and buffer times within the existing timetables. This model was tested on Dutch passenger trains it was shown through the evidence that the slight modifications to the existing timetables can effectively reduce the average delays and some improved results are reported in tables in detail. Liebchen and Stiller^12^ studied both aperiodic (non-periodic) and periodic timetables to ensure the secure journey of passenger’s travel time against a certain amount of lateness. Two heuristics were proposed and thoroughly examined, with a detailed analysis of their respective advantages and disadvantages.

Another variant is the train unit assignment problem (TUAP) defines a list of scheduled train routes, with the number of passenger seats required and a collection of train units each having a specific quantity of available passenger seats. Cacchiani et al.^13^ presents a heuristic approach based on the Lagrangian relaxation for TUAP to tackle the constraints associated with seat requirements. The approach was tested on real-world instances and the obtained results were poor for the case study. For that Later, the same authors^14^ proposed a new fast heuristic algorithms based on Lagrangian relaxation and compared it with the already existing method. These results are illustrated the capacity to achieve favorable solutions within a reasonable amount of time frame.

Jamili et al.^15^ deals with scheduling different types of trains on a single railway track and produced periodic train timetables. A periodic event scheduling problem (PESP) is modeled to generate the primary train timetables. A hybrid meta-heuristic algorithms are based on simulated annealing (SA) and particle swarm optimization (PSO) was developed to find the best solutions to large scale problems, and also experimented with through a case study on an Italian railway.

Another important variant of the TTP is observed on minimizing the passenger waiting time at the stations with additional constraints. Barrena et al.^16^ proposed two mathematical programming formulations for aperiodic train timetables on a single railway track. A fast adaptive large neighborhood search (ALNS) meta-heuristic was developed to find the best solutions to large-scale problems within a short computational time. Niu et al.^17^ focused on the model where train skip-stop patterns are given, established a unified quadratic integer programming approach with linear constraints, which was applied to real-world test instances through numerical experiments, and the obtained some improved results. Further, Jiang et al.^18^ extended the skip stop variant of the TTP with multiple trains by inducting extra stops or skipping certain stops on a double line track. A heuristic method based on Lagrangian relaxation was proposed, tested on various instances taken from the Chinese railway network with atmost 387 trains and obtained the improved train operating schedules.

Shang et al.^19^ focused on system wide equity performance in an overcrowded urban rail transit (URT) network using a multi-commodity flow formulation. The objective was to minimize the overall expenditure by all the passengers within the passenger space time network through the implementation of a skip-stop pattern. A Lagrangian relaxation was proposed to optimize the train skip-stop model and tested on the instances taken from the Beijing rail network, obtained an approximate solution that ensures a minimal gap in the lower bound within the least computational time.

Albrecht et al.^20^ investigated the TTP in the context of track maintenance. A problem space search (PSS) meta heuristic algorithm is designed to determine the minimum sum of train and track maintenance delays. The algorithm efficiently generates a large number of alternative train schedules from which they extract the information on the best operative schedules for the track maintenance. A detailed case study was discussed on a single track rail network in Queensland, Australia. Later, Zhang et al.^21^ proposed a bi-objective optimization model in a railway network, in which the first objective is to minimize the total travel time of passengers and the second objective is to minimize the track maintenance cost. A heuristic algorithm based on Lagrangian relaxation was developed and experimented on the Chinese railway network. The experimental results indicate that the algorithm efficiently determines the best operative train schedules on large-scale rail networks.

Alaghband and Farhang Moghadam^22^ focused on the optimization of freight train scheduling on a single track that aims to minimize trains travel time, allocate freight to freight trains, and reduce the delayness of the trains at the destination. An integer linear programming models are used to represent both scheduling and allocation problems. A novel inspired PSO heuristic algorithm was proposed and experimented on randomly generated data sets to validate the efficiency of the algorithm. The algorithm is capable of solving large scale problems in the railway network.

Zhang et al.^23^ investigated the optimization of both train timetabling and platforming problems, taking into account of various resources such as track segments, station throat areas, and platforms. A mesoscopic perspective is utilized to develop a three-part space time (TPST) framework for designing a train schedule and assigning tracks at stations simultaneously. The objective is to minimize total weighted train running costs. To validate the effectiveness of the model, experiments were conducted on both small and medium sized railway networks, involving the change of some parameters. Detailed reports are provided on the quality of the solution, which was obtained with less computation time. Zhang et al.^24^ addressed the problem of optimizing train timetabling, platforming, and network maintenance simultaneously within a high-speed railway network. A 0–1 integer programming approach was used to model the total weighted train running time and deviations in track maintenance start times. A heuristic method is proposed that dynamically updates the time windows to control the train paths and reduce the solving time for the designed planned trains. These results indicate that the capability of finding high quality solutions fastly within a short amount of time. In the TTP the pilgrim’s departure times are addressed by Owais^25^. A dynamic graph theory formulation is used to minimize the pilgrim’s waiting time at the stations. They incorporate two levels of decisions: the first one involves making real-time decisions, often relying on a greedy method to achieve a quick response and the second one is the decisions are based on learning and are continuously optimized over time.

Some authors studied different models within URT networks. Wang et al.^26^ investigated the TTP in a URT network. The train scheduling includes three types of events: departure and arrivals, fluctuations in passenger arrival rates, and specific routes of passenger arrivals. The objective is to minimize the trains operation cost and the passenger’s total travel time. A sequential quadratic programming (SQP) and genetic algorithm (GA) are proposed and tested for the effectiveness of the algorithms through the case study. The obtained schedules are compared with two existing schedules with a fixed departure headway. The train schedule obtained using the SQP method results in lower energy consumption and ensures more passengers reach their destinations within the time period. The travel time for the passengers that completed their trip obtained by the GA is lower than the obtained within the period by the SQP method.

Later Wu et al.^27^ studied the URT operation based on energy saving operation scheduling technique. A firefly algorithm for urban rail transit operation scheduling (FURTOSO) was developed and tested on the Chengdu metro line. The aim was to decrease the energy consumption and reduce the urban pollution during the operation of URT. To test the performance of this algorithm, it was compared with bacterial foraging optimization (BFO), particle swarm optimization (PSO), and genetic algorithm (GA) through 50 independent runs. The FURTOSO algorithm achieves optimal values in 76 iterations, while the BFO algorithm takes 815 iterations, PSO requires 135 iterations, and GA needs 203 iterations. FURTOSO demonstrates superior performance when compared to the other three algorithms.

Buurman et al.^28^ studied a multi objective problem which is minimizing the difficulties of train operators and maximizing the flexibility for contractors. $\varepsilon -$ constraint method and NSGA-II were proposed and experimented through a case study on the Dutch railway network. The experiments demonstrated that the $\varepsilon -$ constraint method outperforms NSGA-II in identifying superior pareto optimal solutions. The $\varepsilon -$ constraint method is only suitable for smaller size problems and specifically it encountered difficulties network containing more than 15 arcs. A modified shortest path method that considers the limitations of path finding for train traffic supports NSGA-II. Sahebi et al.^29^ was proposed a robust programming model for train movement scheduling that takes into account that dwell intervals at each station and headway between the two successive trains. With the real-world data, a robust optimization approach propose an optimal train schedule that minimizes passenger waiting time and maximizes electrical energy converting from the kinetic energy. A GA is applied to real data from a subway line in the Tehran metro to find the optimal solution.

Very recently, Huang et al.^30^ studied on designing and optimizing train schedules to satisfy the passenger demands. A multi objective programming model is developed to maximize the quality of service provided to passengers and minimize the train operating cost and passenger travel efficiency. The dynamic programming approach is used to simulate the train operation and GA was proposed and experimented on the large and small routes that are dynamically generated. The computational results are indicate that the algorithm effectively solves the practical cases and its train timetable within a short amount of time. Weert et al.^31^ addressed the problem with the aim is to minimize the passenger’s delays events requests from the passenger operators are taken into consider. The simulated annealing meta heuristic is used to find the initial solution and MILP is solved with the $B\&B$ and experimented through a case study on Dutch railway network. The case study demonstrates that scheduling is more convenient to the passenger without wait no longer time.

In addition to this, some more models are discussed on the route network problems in the literature. The bus stop waiting times and bus network design problems are addressed by Owais and Hassan^32^ and Almutairi et al.^33^. Owais et al.^34^ addressed the transit route network design problem as a multi-objective set covering problem. This problem involves three sequential stages that are route generation, route selection, and evaluation (multi-objective analysis). A route constructive genetic algorithm is proposed to generate a large number of candidate routes that corresponds to design objectives. Subsequently, a set covering problem (SCP) is formulated for the selection stage. The algorithm was tested on some benchmark network problems. The results shows that the algorithm is able to produce pareto (or near pareto) optimal solutions. Later, the transit assignment models with a graph formulation are studied by Owais and Ahmed^35^. Now a days travel demand prediction stage is the most important stage in any transportation network. Owais^36^ presents some travel demand models and studied machine learning and micro simulation tools is incorporated in multi-model networks.

It is observed that most of the studies relates on optimizing the train delayness, train operating costs, passenger’s total travel time, and track maintenance cost with single and bi-objective cases under the several constraints with different heuristic and exact methodologies. However, there is a limited attention was given in the context of optimizing the total profit of the trains operating schedule. The present TTP variant contributes to find the feasible train schedule which optimize the total profit within the travel time threshold under the several practical constraints. A case study with two major stations in Indian railways is considered, which looks for the maximum profit within a specified travel time. A $B\&B$ algorithm is developed to find an optimal solution. The enhancement of rail network is inevitable to faster the large scale passenger movements and cargo shipments from one location to another far locations. The risk and air pollution in train traveling is much lower than on road travelling.

The remaining paper is organized as follows: section "Problem description and mathematical formulation" provides a detailed problem description of the TTP and its mathematical formulation. Section "Branch and bound algorithm" presents the discussion on the Branch and bound algorithm. Section "Numerical illustration" includes the numerical illustration of TTP and the extensive computational results are reported in section "Computational experiments" and section "Conclusion" is the concluding remarks of TTP.

## Problem description and mathematical formulation

The Table 1 gives a list of notations used to describe the TTP.

**Table 1:** Notations.

Notations	Description
$n$	Number of stations
$\mathcal{N}$	Node set
$\mathcal{A}$	Arc set
$\phi$	Source station
$d$	Destination station
$m$	Number of trains
$\mathcal{K}$	A set of trains
$\mathcal{P}$	Set of expected profits defined on each arc of $\mathcal{A}$
${P}_{kij}$	The expected profit of train $k$ along the arc ${a}_{ij}$
$\mathcal{T}$	Set of travel times defined on each arc of $\mathcal{A}$
${T}_{kij}$	The travel time of train $k$ along the arc ${a}_{ij}$
${H}_{ki}$	Halting time of train $k$ at the station $i$
${D}_{ki}$	The departure time of train $k$ at the station $i$
${A}_{kj}$	The arrival time of train $k$ at the station $j$
${S}_{k}$	A subset of stations from $\mathcal{N}$ that are visited by the train $k$
$\delta$	Travel time threshold of the train $k$ from source to destination station
$\beta$	The bound value of the terminal node of the branch
${X}_{kij}$	Binary decision variable

Let $\mathcal{G}=(\mathcal{N}, \mathcal{A}, \mathcal{P},\mathcal{T})$ be a general directed acyclic rail network, where $\mathcal{N}$ denotes a node set, $\mathcal{A}$ be an arc set and $\mathcal{P}$ and $\mathcal{T}$ respectively denotes the collection of profit and travel time factors defined on each arc in $\mathcal{A}.$ The node set $\mathcal{N}=\{1, 2, \dots ,n\}$ contains a set of $n$ nodes with source node 1 and destination node $d$, in which each node represents a station. An arc ${a}_{ij}\in \mathcal{A}$ indicate that there exist a train track that connects the station $i$ and station $j.$ Let us consider a set of $m-$ trains say $\mathcal{K}=\{1, 2, \dots , m\}$ be available at the source station and runs from source to destination for moving the passengers/cargos. Additionally, if a train $k$ runs on an arc ${a}_{ij}$ that connects a pair of stations $i$ and $j$, then the non-negative parametric values ${P}_{kij}\in \mathcal{P}$ and ${T}_{kij}\in \mathcal{T}$ respectively defines the expected profit and the expected travel time of train $k$ along the arc ${a}_{ij}.$ In addition to this, when a train departs a station $i$ and arrives a station $j$, then the departure time at station $i (=1, 2, \dots , n-1)$ and the arrival time at station $j(=2, 3, \dots , n)$ of train $k$ respectively denoted as ${D}_{ki}$ and ${A}_{kj}$. The halting time of train $k$ at station $i$ is denoted by ${H}_{ki}, i=1, 2, \dots , n-1.$ The travel time threshold $\delta$ is allowed between the source and destination stations. Furthermore, it is assumed that the trains should start from the source station, can run on several alternative routes (multiple lines) to reach the destination station with the maximum number of passengers passing through a set of intermediate stations. A feasible path of a train $k$ contains a subset of stations ${S}_{k}$ from $\mathcal{N}$ with source and destination stations. Let the cardinality of ${S}_{k}$ be ${\alpha }_{k}$. If a train $k$ is arrived at the station $j$ from station $i$ then the decision variable ${X}_{kij}=1,$ otherwise ${X}_{kij}=0.$ Now, the objective is to determine the best train operating schedules between the source and destination stations which maximizes the profit within the travel time threshold subject to the constraints.

The following assumptions are used to model the TTP.

- The profit between the stations, arrival, departure, and halting timings at the stations are predefined.
- All trains start from the source station and reach the destination station with multiple routes through the intermediate stations.
- Each train enters the station $j$ and departs the station $i$ at most once at the intermediate stations.
- To ensure the safety of trains, the departure and arrival times of any two trains will not be the same the two trains are not coming on the same track in the opposite direction.
- Overtaking is permitted only at the stations.
- Trains do not have a permanent stop at any intermediate stations until they reach their destination.
- All the stations have sufficient platforms for all trains.
  1 $$Maximize \sum_{k=1}^{m}\sum_{i=1}^{n}\sum_{j=1}^{n}{P}_{kij} {X}_{kij}$$

Subject to the constraints

2 $$\sum_{j=1}^{n}{X}_{k\phi j}=1, k\in \mathcal{K}$$

3 $$\left.\begin{array}{c}{\displaystyle{\sum_{j=1}^{n}}}{X}_{kij}\le 1, i\in \mathcal{N}, k\in \mathcal{K}\\ {\displaystyle{\sum_{i=1}^{n}}}{X}_{kij}\le 1, j\in \mathcal{N}, k\in \mathcal{K}\end{array}\right\}$$

4 $$\sum_{i=1}^{n-1}{X}_{yij} {D}_{yi} \ne \sum_{i=1}^{n-1}{X}_{tij} {D}_{ti}, j\in \mathcal{N}, y, t\in \mathcal{K}, y\ne t$$

5 $$\sum_{j=2}^{n}{X}_{yij} {A}_{yj}\ne \sum_{j=2}^{n}{X}_{tij} {A}_{tj}, i\in \mathcal{N}, y, t\in \mathcal{K}, y\ne t$$

6 $${A}_{kj}\le {D}_{kj}, i, j\in \mathcal{N}, k\in \mathcal{K}$$

7 $$\sum_{i\in {S}_{k}}\sum_{j\in {S}_{k}}\left({T}_{kij}+{H}_{ki}\right){X}_{kij} \le \delta , k\in \mathcal{K}$$

8 $$\left| {\sum _{{i \in S_{k} }} X_{{kip}} - \sum _{{j \in S_{k} }} X_{{kpj}} } \right| = \left\{ {\begin{array}{*{20}l} {0,} & {p \ne d,p \in S_{k} ,k \in K} \\ {1,} & {p = 1,d} \\ \end{array} } \right.$$

9 $$2\le \sum_{i\in {S}_{k}}\sum_{j\in {S}_{k}}{X}_{kij}={\alpha }_{k}-1, k\in \mathcal{K}$$

10 $$\sum_{i=1}^{n-1}{X}_{kid}=1, k\in \mathcal{K}$$

11 $${X}_{kij}\in \{0, 1\}$$

The objective function given in (1) looks for maximum profit while operating a set of trains from source station $\phi$ to destination station $d$. Constraint (2) specifies that all trains depart from the source station $\phi$. Constraint (3) denotes that a train $k$ departs from station $i$ and enters station $j$ atmost once. Constraint (4) refers that any two trains departure time should not be same at the source and intermediate stations. Constraint (5) tells that any two trains arrival times should not be same at the intermediate stations and destination station. Constraint (6) denotes that the arrival time of train $k$ at the station $j$ is less than or equal to the departure time of train $k$ at the station $j.$ Constraint (7) represents that the travel time of each train $k$ does not exceed the time threshold value $\delta .$ Constraint (8) preserves the continuity of the path of train $k$ but it does not prevent the occurrence of illegal paths. For example, let ${S}_{k}=${1, 2, 3, 4, 5, 6}. The arcs corresponds to the pair of stations (1, 3), (3, 4), (4, 5), (5, 6) forms a path satisfying the constraints (2)-(8), but station 2 is not included. So, it is an illegal path. To identify the occurrence of such an illegal paths constraint (9) is included. Constraint (9) represents that the path length of train $k$ is bounded between 2 and ${\alpha }_{k}-1$. Constraint (10) says that all trains must arrive to the destination station $d$. A binary decision variable is given in constraint (11).

## Branch and bound algorithm

The branch and bound method is an algorithmic approach used to find the optimal solution for numerous optimization problems, especially in discrete and combinatorial optimization problems. The detailed description of the B&B algorithm is as follows. The B&B carefully examines all the possible permutations implicitly and provides an optimum solution in a sequential manner that starts with an initial node, construct a set of subproblems with branching and eliminates the subproblems which are not feasible or not been further decomposed or not contributing global optima using bounding strategies. The search mechanism of B&B looks like a tree structure. The B&B algorithm can be embedded with different search strategies to speed up the process. The popular search strategies are the depth-first search, breadth-first search, and the best-bound search^37^ etc. The search tree starts with a starting node as a source station $(\phi )$, generating a set of possible nodes through branching that are connecting to $\phi$ in the first state. Each of the connected node pair in the generation represents an arc that links a pair of stations meaning that the existence of train track between the pair of stations. Next, calculate the bounds at the new nodes generated in the tree structure. Let $\beta$ be the best bound value of the terminal node of a branch found so far in the search. Either the bound value of the terminal node is not larger than $\beta$ or the branch is not a partial feasible then discard all nodes that are successors of that node as it cannot contribute to global optimum. Thus, the subtree with that node is rejected implicitly. However, this rejection cannot affect the optimum solution. Further, continue the search in different states recursively by decomposing the branches into sub-branches of smaller size. Each of the sub-branch is regarded as a subproblem. In the process of search if the bound value of the subproblem at a node of the current branch is maximum and is partial feasible when it is compared with its previous bound value then take the current node for further branching or decomposition by neglecting the existing subproblem, otherwise continue to decompose the problem with the existing subproblem.

The complete enumeration of all the subproblems for higher-dimension problems is not practical. Thus, we have to carefully limit the search space without losing the optimal solution. Therefore, the proposed B&B algorithm implicitly enumerates all the possible subproblems along the tree structure and simultaneously stores partial feasible subproblems. Further, the search explores to the uncovered nodes of the solution space iteratively and constructs a complete tree (that starts with the source station and ends with the destination station) with the simpler rules of branching, bounding and termination. Upon generating a complete tree, the algorithm returns with an optimal solution and then the train operating schedules corresponds to the optimal solution will be traced with simple backtracking mechanism. A more recent works on branch and bound can be found in Refs.^38–40^. The limitation of the algorithm is that, it requires a significant amount of memory to explore the entire search for an optimum solution and to store the solution tree, especially for large-size instance problems with too many sub-branches.

In brief, the above framework of B&B involves three important components which have a significant influence on its performance: the first one is search strategy (the sequence in which subproblems within the tree are examined), the second one is branching strategy (how the solution space is partitioned to generate new subproblems within the tree), and the termination (Fathoming) rules (rules that prevent the exploration of suboptimal branches of the tree).

### Proposed branch and bound algorithm


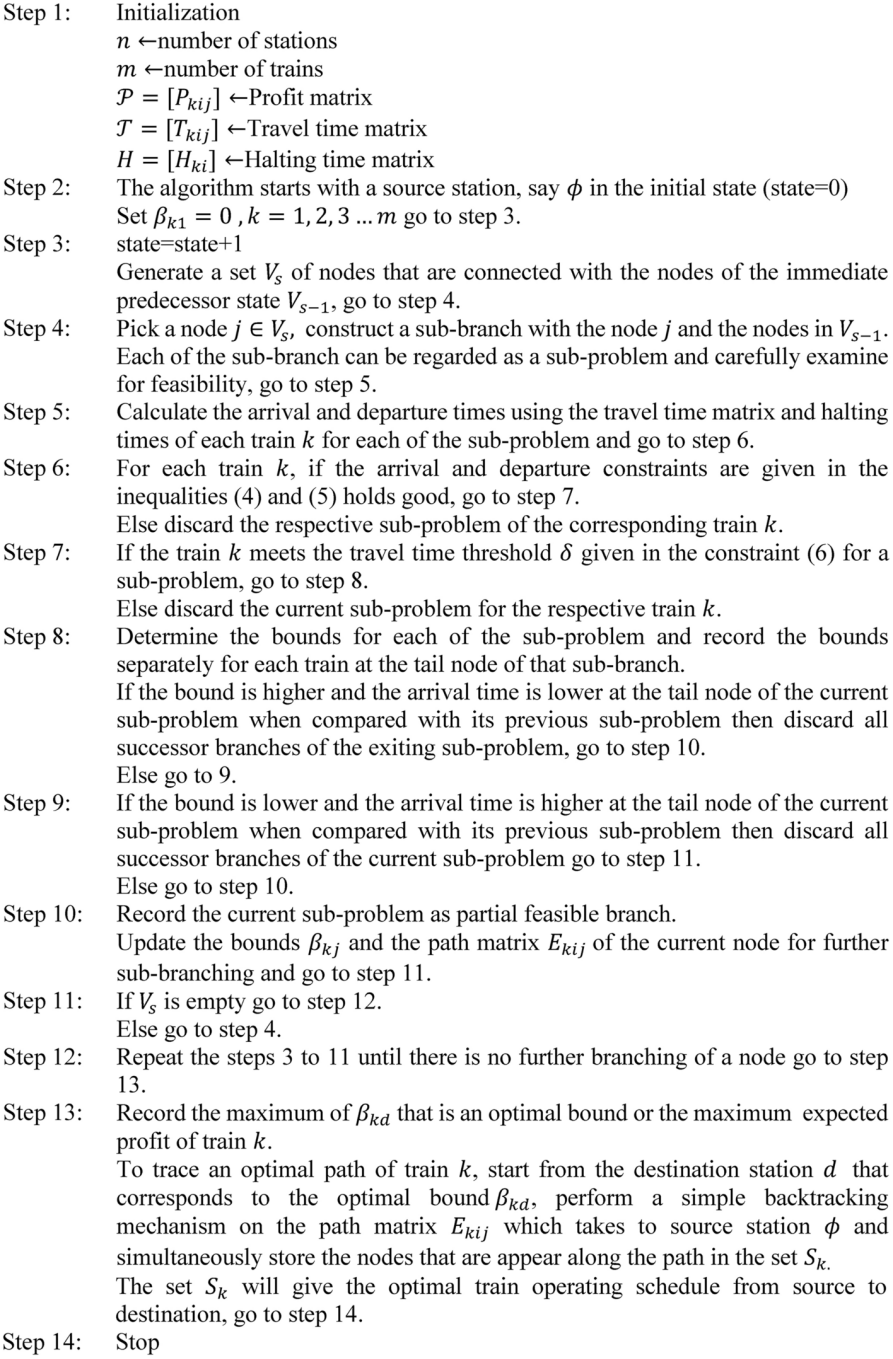


### Flow chart

**Fig. 1:**
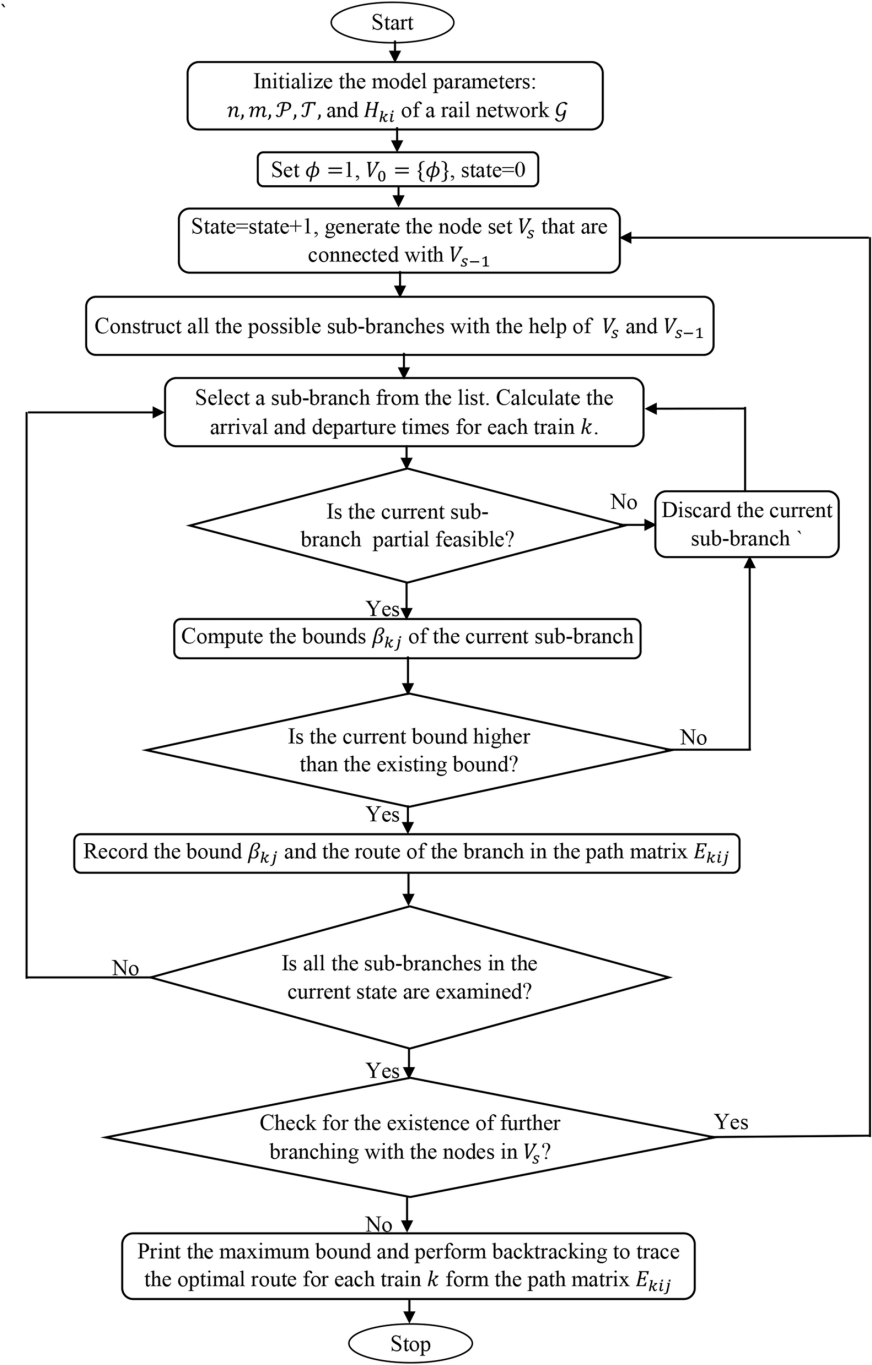
: Flowchart of the proposed B&B.

The systematic procedure of the B&B algorithm is presented in the form of a flow chart in Fig. 1.

## Numerical illustration

**Fig. 2:**
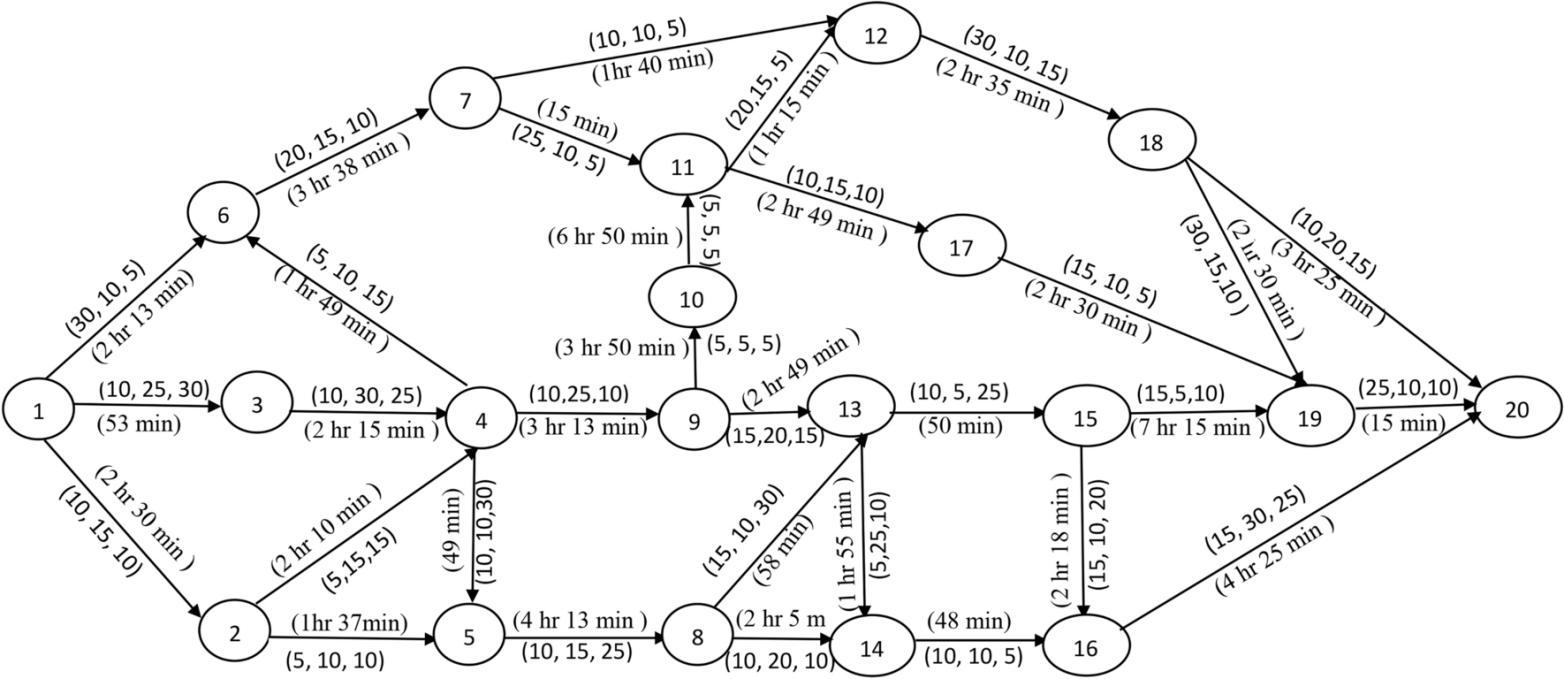
A rail network $\mathcal{G}$ with multiple operative lines between the stations Chennai and Hyderabad.

In this section, the concepts of the proposed TTP is explained by considering a suitable rail network between two prominent Indian metro cities Chennai and Hyderabad. Let the source station $\phi$ be Chennai and the destination station $d$ be Hyderabad. We constructed an appropriate network $\mathcal{G}=(\mathcal{N}, \mathcal{A}, \mathcal{P},\mathcal{T})$ by considering the multiple operative routes (multiple lines) available between the stations Chennai and Hyderabad with a set of intermediate stations/ junctions from an Indian rail network. The network $\mathcal{G}$ contains a total of $\left|\mathcal{N}\right|=n=20$ stations. Let us imagine that there are three trains $(m=3)$ operating between the stations Chennai and Hyderabad which will starts/depart from source station at different times of a day and runs on the network $\mathcal{G}$. The departure time in hours and minutes format of train $k (=1, 2, 3)$ at the source station is ${D}_{k\phi }=\{9:10, 11:25, 12:30\}$. The expected travel times ${T}_{kij}$ of train $k$ between a pair stations $i$ and $j$ and the normalized halting times ${H}_{kj}$ of train $k$ at the station $j$ are taken uniformly from the “Where is my train” application. If the train $k$ departs from station $i$ and arrives to station $j$, then the arrival time of train $k$ at the station $j$ be ${A}_{kj}$, obtained by adding the travel time between the stations $i$ and $j$ to the departure time at station $i$. Thus, ${A}_{kj}$ can be expressed as ${A}_{kj}={D}_{ki}+{T}_{kij}, i=1, 2,\dots , ,n-1, j=2, 3, \dots , n$. Similarly, if the train $k$ arrives to station $j$, then it will be departed by following the specific halting times at station $j$. The departure times at station $j$ can be expressed as ${D}_{kj}={A}_{kj}+{H}_{kj}, j=2, 3, \dots , n$. The arrival times of train $k$ at the source station and the departure times at the destination station are assumed to be zero. The details pertaining to the selected stations such as station name, station code, and the halting times at the stations are given in Table 2. Further, the stations are indexed from 1 to 20, where station 1 and station 20 respectively denote the source and destination stations. The notation ‘–’ used in Table 2 indicate that the train $k$ halts at the destination permanently. On each arc ${a}_{ij}\in \mathcal{A}$, the expected profit ${P}_{kij}\in \mathcal{P}$ and the expected travel time ${T}_{kij}\in \mathcal{T}$ are defined. The information is summarized in the network shown in Fig. 2.

**Table 2:** The expected halting times at the distinct stations.

SN	Station Name	Station Code	Halting Time $\left({H}_{kj}\right)$ in minutes
1	Chennai	MAS	0
2	Katpadi	KPD	5
3	Arakkonam	AJJ	2
4	Renigunta-Tirupati	RU/ TPTY	8
5	Pakala	PAK	2
6	Gudur	GDR	2
7	Tenali	TEL	2
8	Anantapur	ATP	5
9	Kadapa	HX	5
10	Nandyal	NDL	2
11	Guntur	GNT	10
12	Vijayawada	BZA	10
13	Gooty	GY	5
14	Dhone	DHNE	5
15	Guntakal	GTL	5
16	Kurnool City	KRNT	5
17	Nalgonda	NLDA	2
18	Warangal	WL	7
19	Secunderabad	SC	2
20	Hyderabad-Kacheguda	KCG	–

**Fig. 3:**
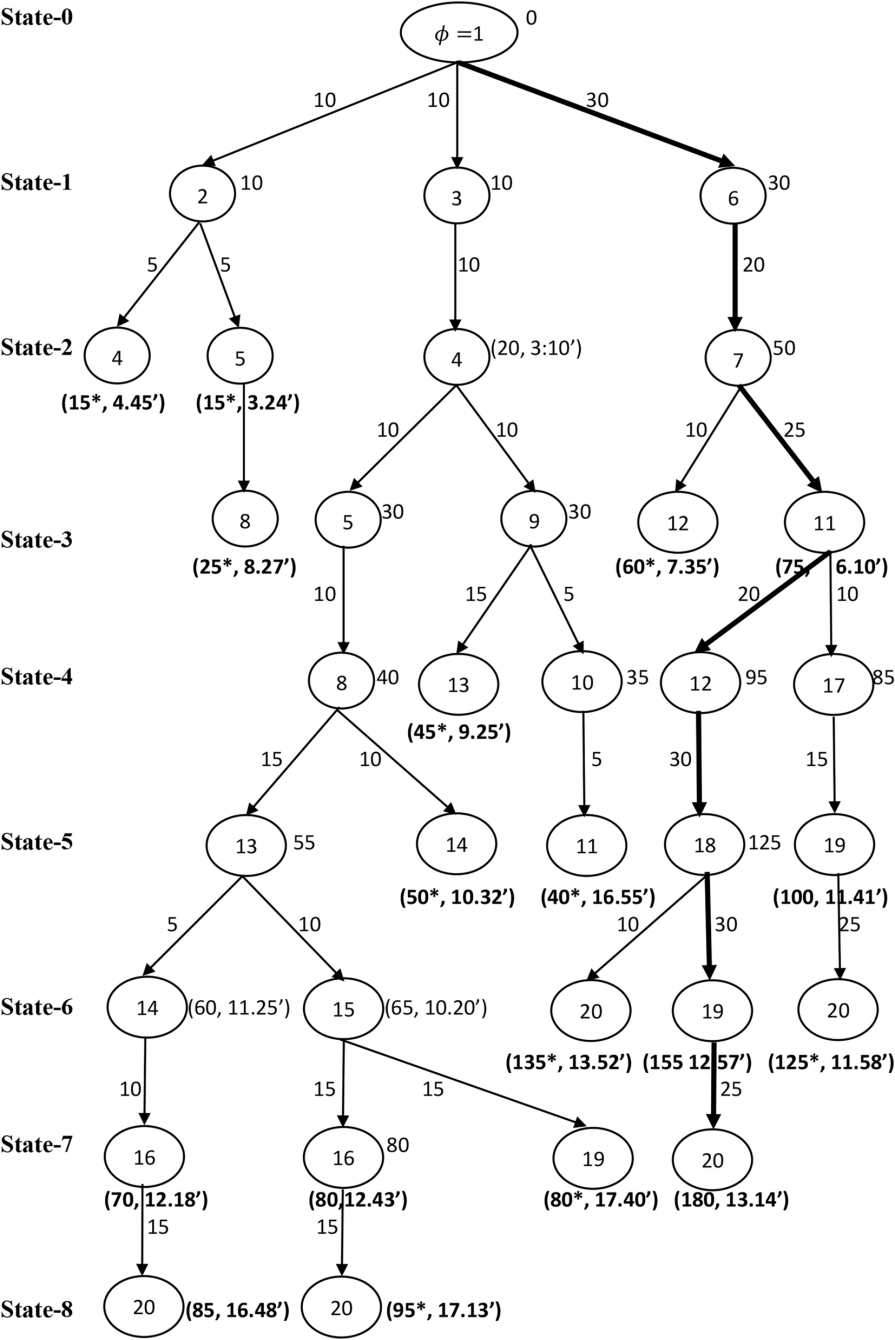
Branching process of train 1.

**Fig. 4:**
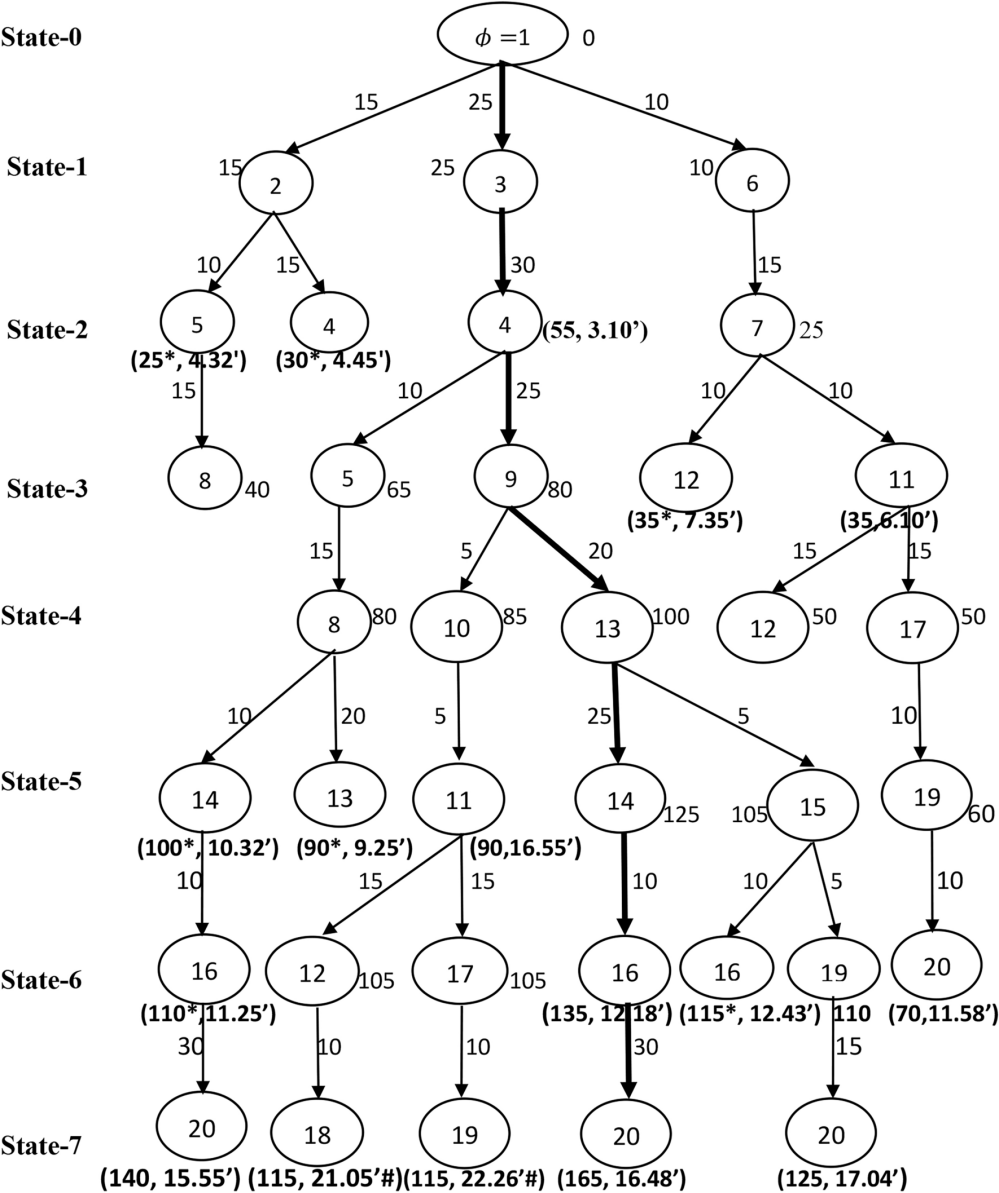
Branching process of train 2.

**Fig. 5:**
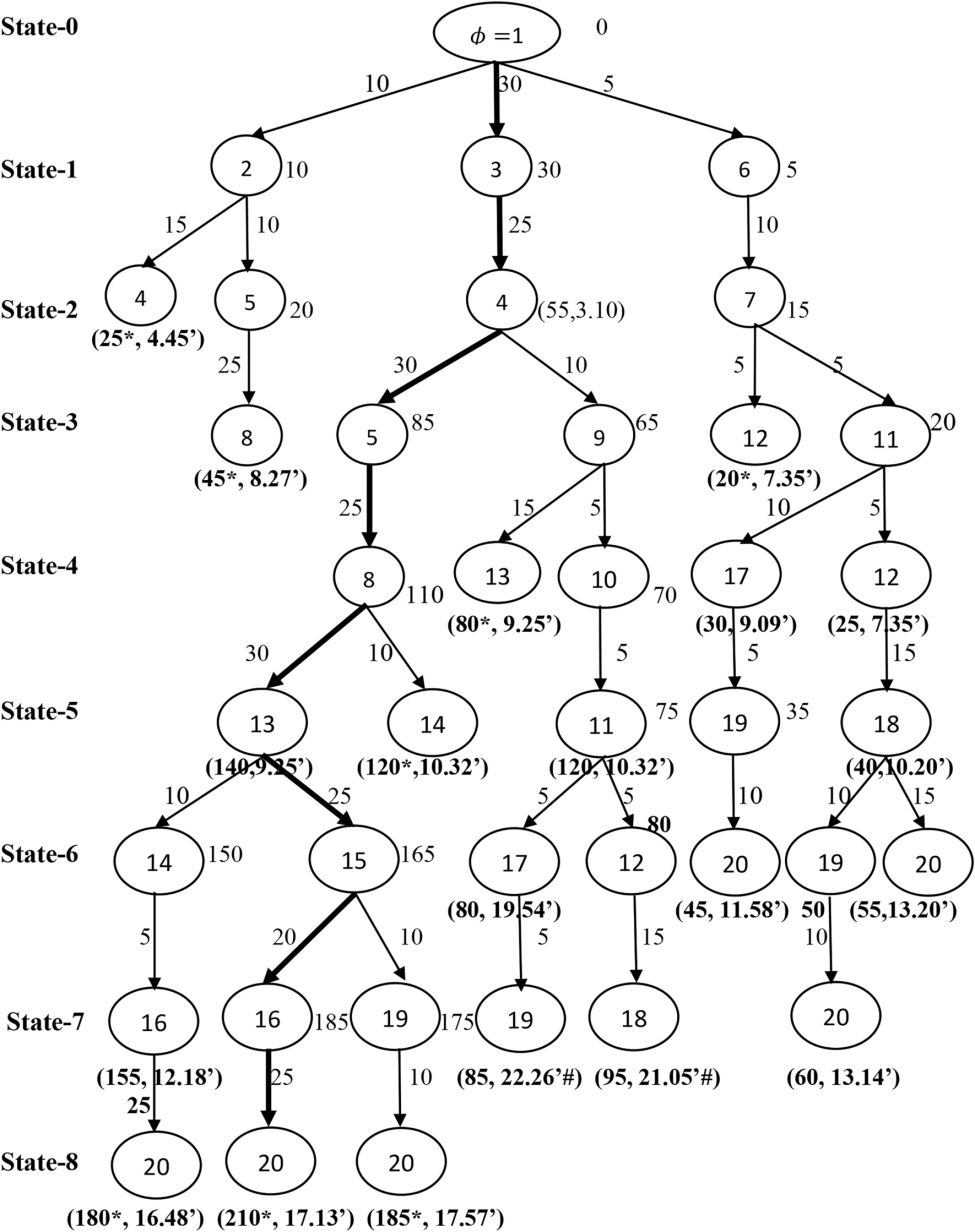
Branching process of train 3.

The expected profits of each train between a pair of stations are generated at random in the interval [5, 30]. The ordered triplet $\left({P}_{1ij}, {P}_{2ij}, {P}_{3ij}\right)$ on an arc ${a}_{ij}\in \mathcal{A}$ in Fig. 2, denote the expected profit of train 1, train 2 and train 3 between the pair of stations $i$ and $j$, respectively. The expected travel times between a pair of stations are defined on an arc ${a}_{ij}\in \mathcal{A}$ are taken in hours and minutes format of Indian standard time of 24 h of a day. In addition, the overall running time of each train between the source station and the destination station should not surpass the travel time threshold $\delta$. Let $\delta =20$ hours. All the trains should start from the source station and reach the destination station with the possible maximum number of passengers/cargos through a set of intermediate stations. Now, the objective is to determine the optimal operative schedules of trains which will give us the maximum total profit. A modified B&B algorithm discussed in Section "Branch and bound algorithm" is used to find an optimal solution of the constructed network $\mathcal{G}$. The algorithm systematically generate and store the feasible sub-problems with the help the effective branching and bounding strategies simultaneously. This systematic search procedure of the proposed B&B shown in the Figs. 3, 4 and 5 with corresponds to the train $k (=1, 2, 3)$ independently for better understanding of readers.

The branching starts with an initial state 0 at the source station $\phi =1$ with an initial bound ${\beta }_{k\phi }=0$. List the stations that are connected with the source station $\phi$ say ${V}_{1}=\left\{2, 3, 6\right\}$. Construct the possible sub-branches with the stations in ${V}_{1}$ and the initial station $\phi$ in state 1. Calculate the bounds and check the feasibility of each of the sub-branch as explained in Section "Proposed branch and bound algorithm". The calculated bounds are projected at each of the tail node of the respective sub-branch. Next, continue to generate a set of stations say ${V}_{2}=\left\{4, 5, 7\right\}$ that are connected with the stations in ${V}_{1}$ in state 2 and followed calculate the bounds and check for feasibility of the current sub-branches. Record the latest bounds at the tail nodes of each of the current sub-branch. In similar manner, continue the search until there is no further branching.

### Infeasible and optimal solutions

Figure 3, explains the detailed branching and bounding process of determining the best operative schedule for train 1. Note that, two cases arises in fathoming a sub-branch. In the first case if a tail node connected with two sub-branches then fathom a sub-branch which has low profit with higher travel time, as it cannot produce optima, indicated as ${^{\prime}}*{^{\prime}}$ and in the second case fathom the sub-branch whose travel time is more than $\delta$, as it is infeasible, indicated as ${^{\prime}}\#{^{\prime}}$. For example, in Fig. 3, observe that the bound at the tail node 4 along the sub-branch $1\to 2\to 4$ is 15 units of profit with the travel time 4 h 03 min, whereas the sub-branch $1\to 3\to 4$ contributes the profit 20 units with the travel time 3 h 10 min. Therefore, the sub-branch $1\to 2\to 4$ fathomed due to the low profit with higher travel time at the tail node 4. The expected profit and the travel time at the tail node of the fathomed sub-branch is represented as an ordered pair. The sub-branch $1\to 3\to 4$ will be stored for further branching with the bound at the tail node 4 as $20$ units. Consider the sub-branch $1\to 6\to 7\to 11\to 17\to 19$, that produce 100 units of profit with 11 h 41 min of travel time, while the sub-branch $1\to 6\to 7\to 11\to 12\to 18\to 19$ produce 155 units of profit with 12 h 57 min of travel time. Although the sub-branch $1\to 6\to 7\to 11\to 12\to 18\to 19$ give the higher profit than the sub-branch $1\to 6\to 7\to 11\to 17\to 19$, and it may be discarded in a future state with infeasibility or the case of exceeding the time threshold $\delta$. Hence, do not fathom the sub-branch $1\to 6\to 7\to 11\to 17\to 19$ at its current state as there may be a chance that it can appear in the solution due its low travel time. Further, the search is continued till the destination station is arrived. Figure 4 show the branching process for train 2. Observe from Fig. 4, Train 2 takes 21 h 05 min of travel time along the sub-branch $1\to 3\to 4\to 9\to 10\to 11\to 12\to 18$. Thus, this sub-branch is fathomed due to the violation of the travel time threshold $\delta$.

**Fig. 6:**
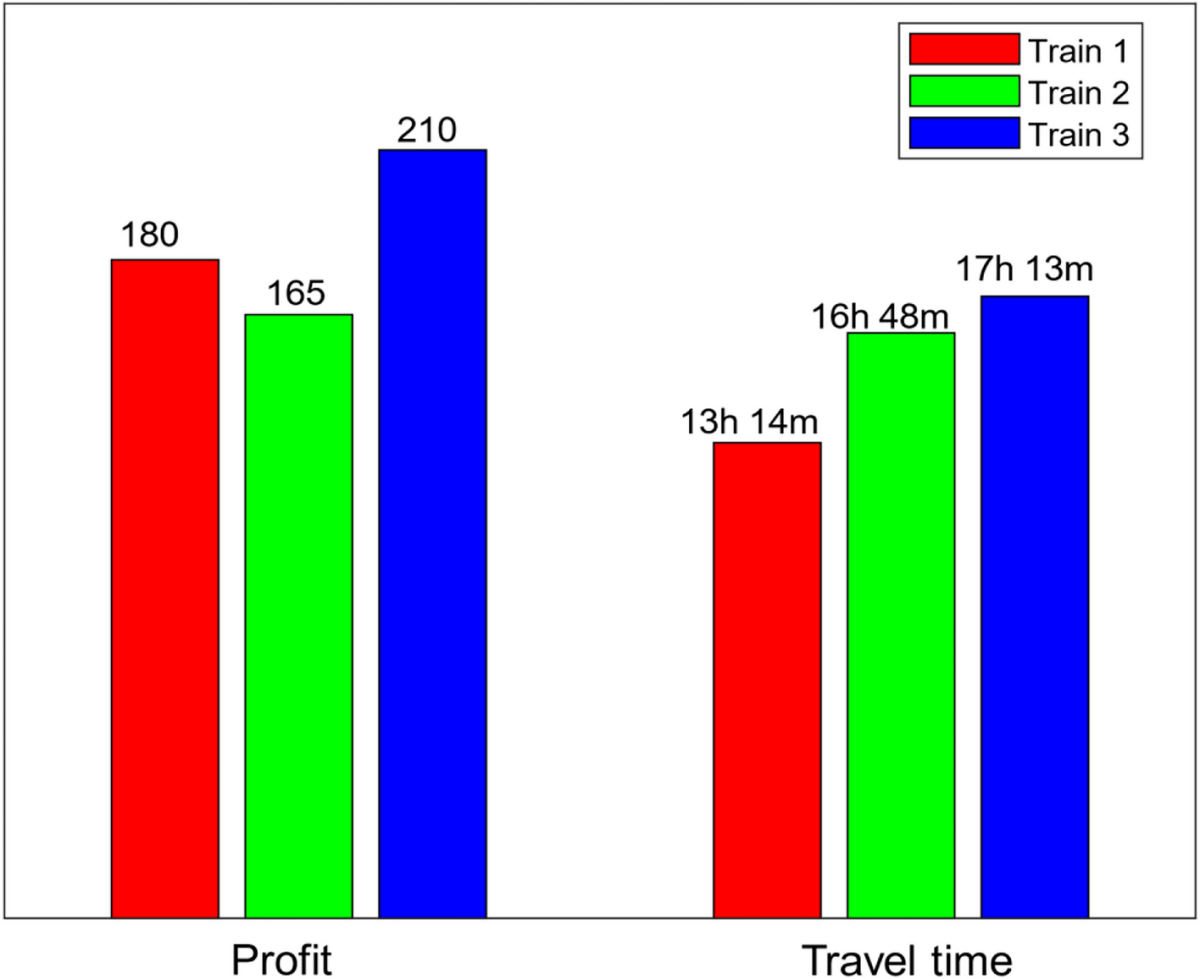
A comparative bar plot of travel time and profit of the three trains.

The set of implicitly enumerated feasible operative routes through the systematic B&B algorithm for the trains 1, 2 and 3 observed from Figs. 3, 4 and 5 are summarized in Table 3. The columns 1 through 5 in Table 3, respectively denotes the train index, feasible operative routes of train $k, k=1, 2, 3$, the number of stations ($\#{m}_{1}$) involved in each of the feasible route, the consumption of travel time and the profit generated on the respective route. Note that, there are five feasible routes for train 1 from Chennai to Hyderabad with varied profit and travel time, in which the route $1\to 6\to 7\to 11\to 17\to 19\to 20$ takes least travel time 11 h 58 min with the profit 125 units. Another route $1\to 6\to 7\to 11\to 12\to 18\to 19\to 20$ makes 180 units of profit with travel time 13 h 14 min. Since, the objective is to find a route with maximum profit, thus the route $1\to 6\to 7\to 11\to 12\to 18\to 19\to 20$ is selected as best operative route for train 1. Thus ${S}_{1}=\{1, 6, 7, 11, 12, 18, 19, 20.\}$. The train $-$ 2 have three distinct feasible solutions within the travel time threshold $\delta$. The route $1\to 3\to 4\to 9\to 13\to 14\to 16\to 20$ generates maximum profit 165 units with travel time 16 h 48 min among other routes and is regarded as best operative route for train 2. Thus ${S}_{2}=\left\{1, 3, 4, 9, 13, 14, 16, 20\right\}.$ As many as seven feasible routes for the train 3 within $\delta$, among all those routes, the route $1\to 3\to 4\to 5\to 8\to 13\to 15\to 16\to 20$ is considered as best operative route with 210 units of profit and 17 h 13 min travel time. Thus ${S}_{3}=\left\{1, 3, 4, 5, 8, 13, 15, 16, 20\right\}.$ Therefore, the optimal profit or total maximum profit generated by all the three trains on the given network within the time threshold $\delta =20$ hours is 555 units and the optimal routes of the three trains covers 18 intermediate stations between the stations Chennai and Hyderabad. In addition, the maximum profits and the travel times observed for the three trains corresponds to the best operative routes is shown through a comparative bar plot in Fig. 6. Train 3 makes greatest profit among all the three trains considered. However, train 3 travel time between the two metro stations is higher than the other two trains travel time.

**Table 3:** The detailed feasible operative routes for the trains.

Train type	Feasible routes of the trains	$\#{m}_{1}$	Travel time	Profit
Train $-$ 1	$1\to 3\to 4\to 5\to 8\to 13\to 14\to 16\to 20$	9	16 h 48 m	85
	$1\to 3\to 4\to 5\to 8\to 13\to 15\to 16\to 20$	9	17 h 13 m	95
	$1\to 6\to 7\to 11\to 17\to 19\to 20$	7	11 h 58 m	125
	$1\to 6\to 7\to 11\to 12\to 18\to 20$	7	13 h 52 m	135
	$1\to 6\to 7\to 11\to 12\to 18\to 19\to 20$	8	13 h 14 m	180
Train $-$ 2	$1\to 6\to 7\to 11\to 17\to 19\to 20$	7	11 h 58 m	70
	$1\to 3\to 4\to 9\to 13\to 15\to 19\to 20$	8	17 h 04 m	125
	$1\to 3\to 4\to 9\to 13\to 14\to 16\to 20$	8	16 h 48 m	165
Train $-3$	$1\to 6\to 7\to 11\to 17\to 19\to 20$	7	11 h 58 m	45
	$1\to 6\to 7\to 11\to 12\to 18\to 20$	7	13 h 20 m	55
	$1\to 6\to 7\to 11\to 12\to 18\to 19\to 20$	8	13 h 14 m	60
	$1\to 3\to 4\to 5\to 8\to 14\to 16\to 20$	8	15 h 55 m	140
	$1\to 3\to 4\to 5\to 8\to 13\to 14\to 16\to 20$	9	16 h 48 m	180
	$1\to 3\to 4\to 5\to 8\to 13\to 15\to 19\to 20$	9	17 h 57 m	185
	$1\to 3\to 4\to 5\to 8\to 13\to 15\to 16\to 20$	9	17 h 13 m	210

Train 1 starts from the source station Chennai at 9:10, runs through its optimal route, arrives the next station Gudur at 11:23 and departs after 2 min of halt, later arrives Vijayawada at 16:45 and halts 10 min, and finally reaches the destination station Hyderabad at 22:24. Train 2 may starts from Chennai at 11:25 reaches Hyderabad at 4:13 next day. Train 3 may start from Chennai at 12:30 reaches Hyderabad at 5:43 next day. The detailed arrival and departure schedules at various stations of the optimal operative routes for the three trains provided in Table 4. If we set the time threshold value as $\delta =15$ hours, then the optimal profit within the $\delta$ is found to be 310 units from the list of operative routes given in Table 3 and this profit is reduced by more 40% of the previous optimal profit. Further, the time threshold $\delta$ is reduced to $12$ hours, then the optimal profit is observed to be 240 units from Table 3 and this solution is much lowered than the earlier solutions. Note that, the $\delta$ can significantly influence the search region and an optimal solution.

**Table 4:** The expected arrival and departure times of trains at distinct stations.

SN	Station Name	Station code	Train 1		Train 2		Train 3	
			Arrival time ${(A}_{1j})$	Departure time ${(D}_{1j})$	Arrival time $({A}_{2j})$	Departure time $({D}_{2j})$	Arrival time $({A}_{3j})$	Departure time ${(D}_{3j})$
1	Chennai	MAS	0	9:10	0	11:25	0	12:30
2	Katpadi	KPD	–	–	–	–	–	–
3	Arakkonam	AJJ	–	–	12:18	12:20	13:23	13:25
4	Renigunta/ Tirupati	RU\ TPT	–	–	14:35	14:43	15:40	15:48
5	Pakala	PAK	–	–	–	–	16:37	16:39
6	Gudur	GDR	11:23	11:25	–	–	–	–
7	Tenali	TEL	15:03	15:05	–	–	–	–
8	Anantapur	ATP	–	–	–	–	20:52	20:57
9	Kadapa	HX	–	–	17:56	18:01	–	–
10	Nandyal	NDL	–	–	–	–	–	–
11	Guntur	GNT	15:20	15:30	–	–	–	–
12	Vijayawada	BZA	16:45	16:55	–	–	–	–
13	Gooty	GY	–	–	20:50	20:55	21:55	22:00
14	Dhone	DHNE	–	–	22:50	22:55	–	–
15	Guntakal	GTL	–	–	–	–	22:50	22:55
16	Kurnool City	KRNT	–	–	23:43	23:48	01:13	01:18
17	Nalgonda	NLDA	–	–	–	–	–	–
18	Warangal	WL	19:30	19:37	–	–	–	–
19	Secunderabad	SC	22:07	22:09	–	–	–	–
20	Hyderabad- Kacheguda	KCG	22:24	0	04:13	0	05:43	0

**Fig. 7:**
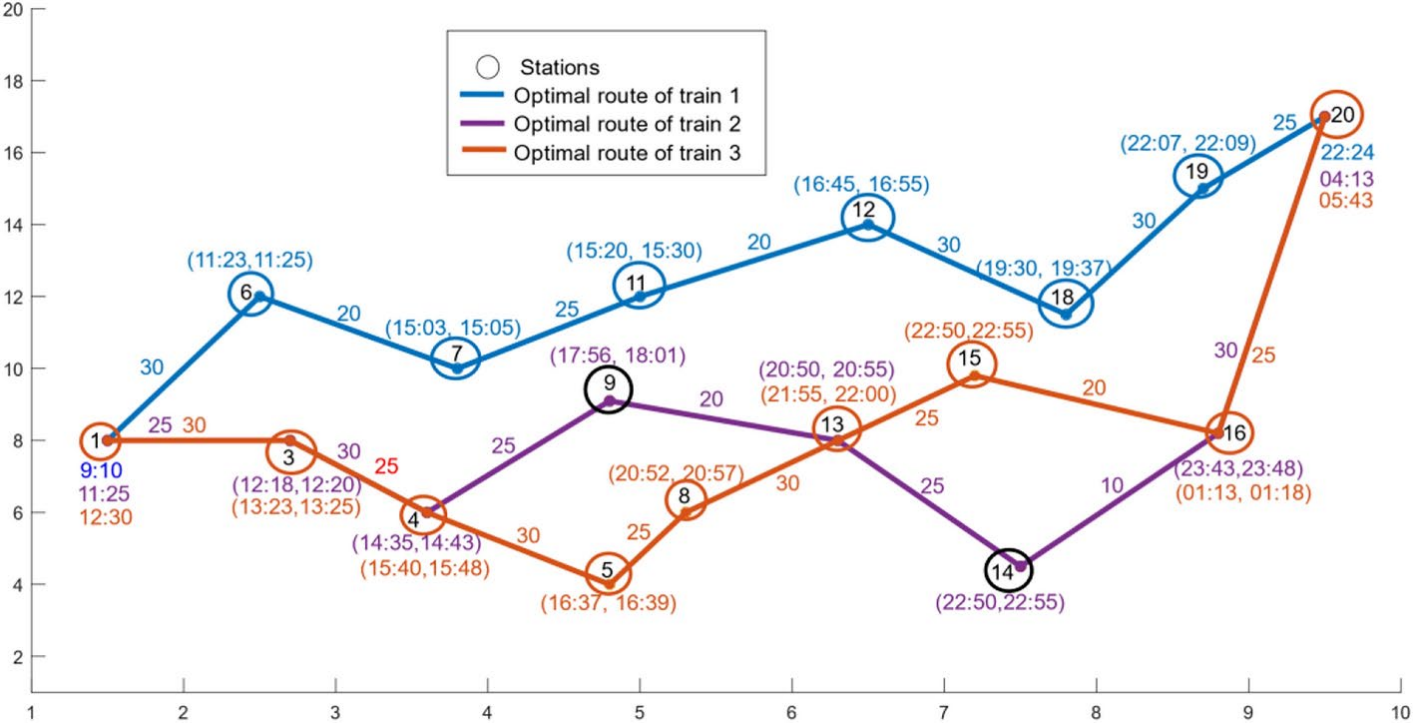
An optimal schedule of three trains from source to destination stations.

The optimal operative routes for the three trains that are connected with the two metro stations Chennai and Hyderabad is shown graphically in Fig. 7. The stations are scattered on a Euclidian plane. An ordered pair $\left({A}_{kj}, {D}_{kj}\right)$ at the station node represents an arrival and departure times of trains, ${D}_{k\phi }$ and ${A}_{kd}$ represents the departure time of trains at the source station and arrival time of trains at the destination stations respectively. The profit ${P}_{kij}$ generated by the train $k$ between a pair of stations $i$ and $j$ is projected on an arc that connects stations $i$ and $j.$ It is observed that a few intermediate stations are in common in the optimal routes of train 2 and train 3.

## Computational experiments

This section provides the computational results of TTP with the proposed $B\&B$ algorithm. The algorithm is coded in MATLAB and tested on a PC with 1.00 GHz Intel (R) Core (TM) i5-1035G1 CPU and 4 GB of RAM running the Microsoft Windows 10 operating system. The performance of $B\&B$ tested by generating the distinct test cases in MATLAB. The test cases are classified based on the number of stations $(n)$ are ranging from 25 to 100 stations and the number of trains $(m)$ are ranging from 3 to 10 trains. The profit and the travel time (minutes) between the stations are randomly generated in [10, 80] and [15, 90], respectively. The halting time (minutes) at the stations are randomly generated in the interval [5, 10]. For each random number generation, a uniform distribution is used in MATLAB.

The experiments are carried out on a class of test instances by varying the values of $n$ and $m$. For readers clear understanding, the complete information of each test case and their solutions are given in Table 5. In Table 5, $SN$ denote the serial number, $n$ represents the number of stations in the test case, distinct trains involved in test case, ${D}_{k\phi }$ and ${A}_{kd}$ respectively represents the expected departure and arrival times at the source and destination stations, $\delta$ be the travel time threshold in hours, $\#{m}_{1}$ be the number of stations appear in an optimal route of train $k$, the travel time of train $k$ from the source to destination station, the profit generated by train $k$ between the source and destination stations and finally $T(s)$ gives the CPU runtime.

**Table 5:** Experimental results on randomly generated instances.

$SN$	$n$	Train type	${D}_{k\phi }$	${A}_{kd}$	$\delta$	$\#{m}_{1}$	Travel time	profit	$T(s)$
1	29	T1	2:05	23:24	24	27	21 h 19 m	1382	0.34
		T2	4:15	26:54		28	22 h 39 m	1416	
		T3	5:45	23:14		20	17 h 29 m	1096	
		T4	6:25	25:38		23	19 h 13 m	1093	
		T5	7:10	27:08		25	19 h 58 m	1055	
2	35	T1	3:00	32:04	30	31	29 h 4 m	1436	0.57
		T2	3:50	28:32		30	24 h 42 m	1373	
		T3	4:20	30:55		33	26 h 35 m	1595	
		T4	5:05	33:53		34	28 h 48 m	1654	
		T5	6:45	29:10		27	22 h 25 m	1441	
3	55	T1	6:00	56:14	51	48	50 h 14 m	2422	0.71
		T2	6:50	56:25		48	49 h 35 m	2626	
		T3	7:35	54:40		51	47 h 5 m	2534	
		T4	8:10	56:31		49	48 h 21 m	2489	
		T5	8:45	58:33		48	49 h 48 m	2433	
		T6	9:30	59:30		49	50 h	2285	
		T7	8:30	60:20		52	51 h 50 m	2685	
4	65	T1	3:15	57:33	55	59	54h18m	2718	0.77
		T2	4:00	54:51		53	50 h 51 m	2889	
		T3	4:40	59:40		57	55 h	2979	
		T4	5:10	55:34		52	50 h 24 m	2573	
		T5	6:00	60:51		59	54 h 51 m	2903	
		T6	6:45	59:37		54	52 h 52 m	2682	
5	75	T1	4:00	60:17	60	60	56 h 17 m	3338	1.32
		T2	4:50	62:08		62	57 h 18 m	2423	
		T3	5:30	60:24		64	56 h 54 m	3272	
		T4	6:10	66:01		64	59 h 51 m	3162	
		T5	7:00	64:32		65	57 h 32 m	3067	
		T6	7:40	67:24		65	59 h 44 m	3234	
		T7	8:15	65:18		66	57 h 3 m	3227	
6	80	T1	6:00	58:56	61	62	52 h 56 m	3318	1.30
		T2	6:45	67:41		67	60 h 56 m	3359	
		T3	7:40	65:50		64	58 h 10 m	3281	
		T4	8:30	69:20		68	60 h 50 m	3586	
		T5	9:15	65:18		70	56 h 13 m	3007	
		T6	10:00	70:25		66	60 h 25 m	3267	
		T7	10:45	71:21		66	60 h 36 m	3424	
7	85	T1	5:00	66:01	65	79	61 h 1 m	3908	1.55
		T2	5:40	69:27		77	63 h 47 m	4013	
		T3	6:15	71:06		77	64 h 51 m	3924	
		T4	7:00	71:17		74	64 h 17 m	3975	
		T5	7:45	70:43		75	62 h 58 m	3790	
		T6	8:30	73:10		74	64 h 40 m	3720	
		T7	9:10	72:29		78	63 h 19 m	4054	
		T8	10:00	72:32		74	62 h 32 m	3948	
8	90	T1	2:30	63:56	65	78	61 h 26 m	3720	1.88
		T2	3:10	65:40		76	62 h 30 m	4115	
		T3	4:00	68:56		74	64 h 56 m	3989	
		T4	4:50	69:23		73	64 h 33 m	3979	
		T5	5:20	68:09		73	62 h 49 m	3865	
		T6	6:00	70:06		75	64 h 6 m	3951	
		T7	7:00	66:07		77	59 h 7 m	3941	
		T8	7:45	70:45		75	63 h	3652	
9	95	T1	2:00	64:27	68	76	62 h 27 m	3889	2.31
		T2	2:40	70:32		80	67 h 52 m	3908	
		T3	3:20	69:56		76	66 h 36 m	4009	
		T4	4:00	70:26		79	66 h 26 m	4074	
		T5	4:50	70:53		79	66 h 3 m	4133	
		T6	5:30	73:23		81	67 h 53 m	4035	
		T7	6:15	73:08		77	66 h 53 m	3946	
		T8	7:00	74:30		75	67 h 30 m	4124	
		T9	7:35	74:54		74	67 h 19 m	3892	
		T10	8:30	76:22		83	67 h 52 m	4244	
10	100	T1	3:30	72:02	72	82	69 h 2 m	4268	2.08
		T2	4:15	74:18		83	70 h 3 m	4261	
		T3	5:00	76:12		84	71 h 12 m	4384	
		T4	5:45	77:32		84	71 h 47 m	4172	
		T5	6:30	77:25		82	70 h 55 m	4343	
		T6	7:10	79:10		80	72 h	4078	
		T7	7:50	76:33		77	68 h 43 m	3906	
		T8	8:20	79:03		85	70 h 43 m	4599	
		T9	8:55	80:34		83	71 h 39 m	4151	
		T10	9:30	81:09		83	71 h 39 m	4170	

The observations on Table 5 are given as follows.

**Fig. 8:**
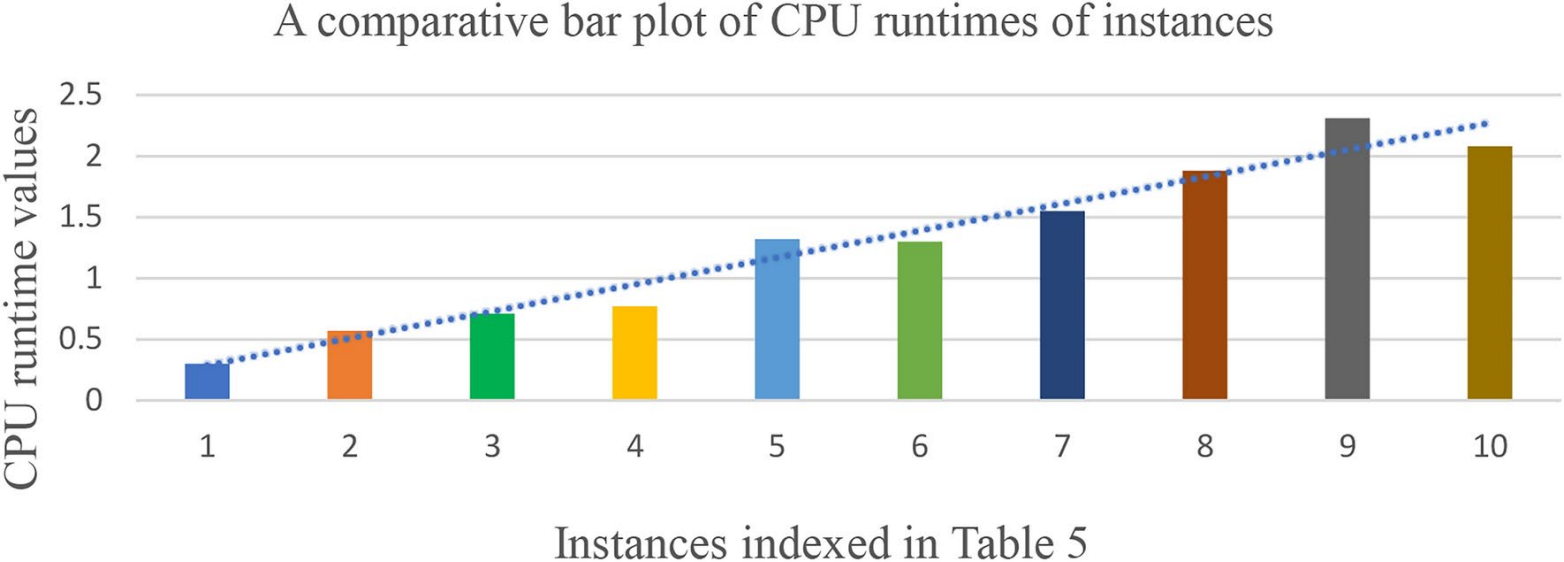
A comparative bar plot of CPU run times of different test instances in Table $5$.

- The algorithm carefully examines the existence of feasible branches, eliminates unwanted branches with the help of effective bounding strategies.
- The results indicate that the algorithm is capable to solve the higher dimension instances of TTP optimally within a reasonable computational time.
- The solution of the algorithm highly influenced by the parametric values $n, m, {P}_{kij}, {T}_{kij}, \delta$.
- When the parametric values $n$ and $m$ increases the total profit and the computational time increasing.
- In each test case, the total travel time and the total profit may differs significantly across the various trains.
- The travel time threshold is taken up to three days of continuous journey.
- The algorithm is capable to obtain the alternate optimum routes of TTP, if exists.
- Fig. 8 depicts the comparative bar plot of CPU runtimes of a total of 10 different test cases given in Table 5.
- From Fig. 8 the overall trends of CPU runtime indicate that the runtime increases as the parametric values $n$ and $m$ increases.

**Fig. 9:**
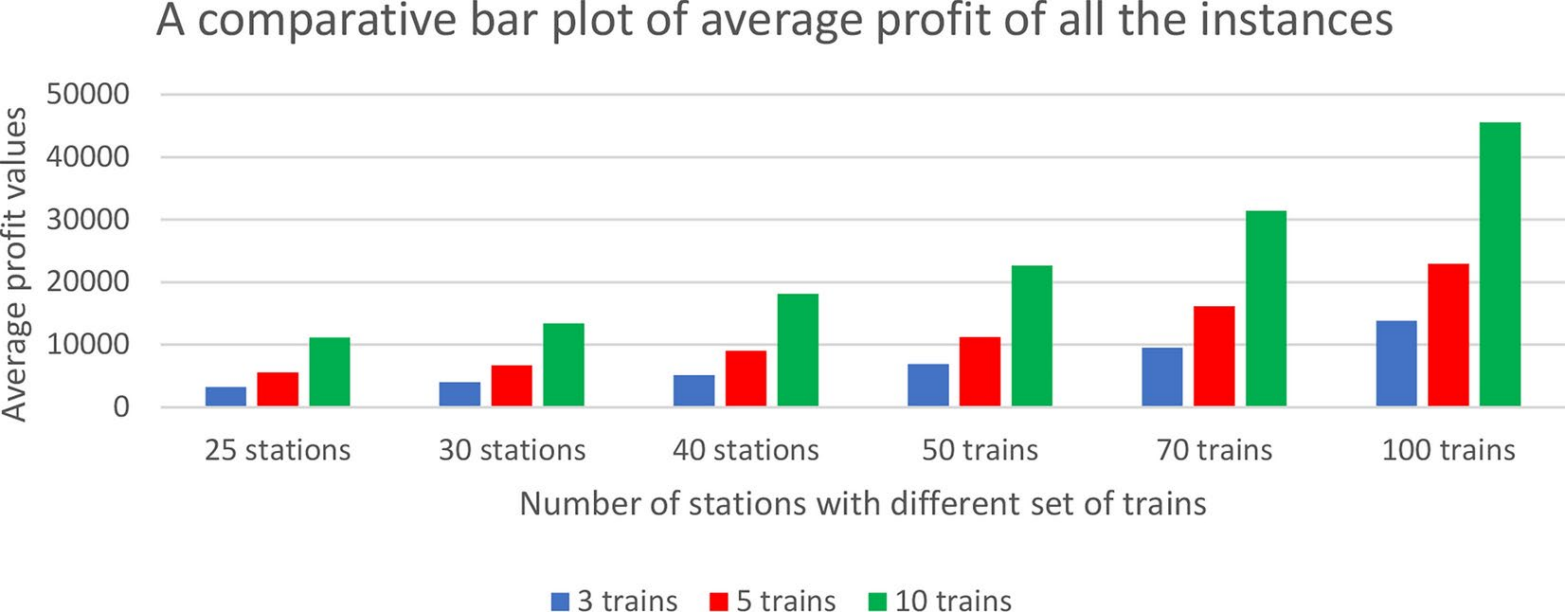
A comparative bar plot of the average profit values.

Further, the descriptive statistical analysis on the performance of the proposed B&B is provided in Table 6. We experimented 18 test cases which are randomly generated by setting different values for $n$, $m$ and $\delta$ in the earlier specified intervals. In each test case we performed 5 independent runs and results a total of 90 instances. For each test case with 5 independent runs, we recorded the minimum, maximum, average and standard deviation in the optimal solutions were obtained and as well the average travel time per train and CPU runtimes. The optimal solutions are highly dependent on the profit and travel time matrices. The larger value of standard deviation indicating that the optimal solutions obtained in those five runs within each of the testcase were widely spread in the solution space. All the optimal solutions are obtained in the less amount of CPU runtime. A comparative bar plot of the average profit of all the instances from Table 6 is shown in Fig. 9.

**Table 6:** Descriptive statistical analysis of B&B.

$n$	$m$	$\delta$	Minimum	Maximum	Average	Standard deviation	Average travel time	Average CPU runtime
25	3	24	3109	3332	3212	81.19	15 h 42 m	0.19
	5		5100	5829	5596	293.61	16 h 27 m	0.20
	10		10,830	11,387	11,171	310.08	16 h 04 m	0.17
30	3	24	3915	4180	4015	104.20	19 h 57 m	0.16
	5		6481	6958	6691	185.17	20 h 07 m	0.15
	10		13,086	13,754	13,446	237.50	19 h 30 m	0.24
40	3	30	4907	5437	5163	221.95	26 h 06 m	0.18
	5		8867	9306	9062	206.03	26 h 22 m	0.18
	10		17,930	18,504	18,116	225.11	26 h 36 m	0.34
50	3	40	6786	7028	6889	103.09	35 h 29 m	0.22
	5		10,849	11,604	11,238	275.40	33 h 36 m	0.34
	10		22,253	22,988	22,645	349.99	33 h 16 m	0.53
70	3	60	9408	9892	9555	201.53	56 h 44 m	0.45
	5		15,878	16,689	16,169	330.72	46 h 25 m	0.54
	10		28,552	32,884	31,404	1687	45 h 57 m	0.94
100	3	70	13,599	14,409	13,833	326.16	66 h 57 m	0.67
	5		22,612	23,195	22,915	225.60	65 h 31 m	1.10
	10		45,040	46,416	45,577	528.5	66 h 02 m	2.07

## Conclusion

This paper presents a mathematical model of TTP, aims to find an optimal operative schedules for trains which runs between a pair of any two major stations. This TTP variant is mathematically formulated with 0–1 integer linear programming. The operating schedules of TTP provides a routing plan and the train’s arrival and departure times at the stations. The TTP have applications in different areas includes air traffic control, logistics and supply chain management, etc. This study contributes a $B\&B$ algorithm which carefully examine all the branches, eliminates the unwanted branches efficiently and obtain the best operative schedules for TTP. The concepts of TTP and $B\&B$ algorithm was explained by considering the rail network between two major Indian metro stations Chennai and Hyderabad. The extensive computational experiments carried out on distinct randomly generated test instances indicate that the algorithm is capable to find the optimal solutions and the descriptive statistical analysis on the performance of B&B algorithm show that the algorithm takes fairly less amount of CPU runtimes. The TTP can be extended to study with multiple objectives, skip-stop plans, rail freight transportation, passenger seat availability and allocations etc. In addition, the formulation of new search strategies in B&B by integrating with evolutionary algorithms can be developed to faster the algorithm in providing the optimal solutions.

## Data Availability

The datasets are used during this study are available from the corresponding author on request.
